# Understanding Loneliness in Younger People: Review of the Opportunities and Challenges for Loneliness Interventions

**DOI:** 10.2196/45197

**Published:** 2023-11-02

**Authors:** Hurmat Ali Shah, Mowafa Househ

**Affiliations:** 1 Hamad bin Khalifa University Doha Qatar

**Keywords:** health informatics, loneliness informatics, loneliness theory, health effects, loneliness interventions, information and communication technology, ICT-based interventions, social-media–based interventions, social media, ICT, lonely, loneliness, social isolation, mental health, psychological

## Abstract

Loneliness affects the quality of life of people all around the world. Loneliness is also shown to be directly associated with mental health issues and is often the cause of mental health problems. It is also shown to increase the risk of heart diseases and other physical illnesses. Loneliness is studied both from the social and medical sciences perspectives. There are also interventions on the basis of health informatics, information and communication technologies (ICTs), social media, and other technological solutions. In the literature, loneliness is studied from various angles and perspectives ranging from biological to socioeconomical and through anthropological understandings of technology. From the ICT and technological sides, there are multiple reviews studying the effectiveness of intervention strategies and solutions. However, there is a lack of a comprehensive review on loneliness that engulfs the psychological, social, and technological studies of loneliness. From the perspective of loneliness informatics (ie, the application of health informatics practices and tools), it is important to understand the psychological and biological basis of loneliness. When it comes to technological interventions to fight off loneliness, the majority of interventions focus on older people. While loneliness is highest among older people, theoretical and demographical studies of loneliness give a U-shaped distribution age-wise to loneliness; that is, younger people and older people are the demographics most affected by loneliness. But the strategies and interventions designed for older people cannot be directly applied to younger people. We present the dynamics of loneliness in younger people and also provide an overview of the technological interventions for loneliness in younger people. This paper presents an approach wherein the studies carried out from the perspectives of digital health and informatics are discussed in detail. A comprehensive overview of the understanding of loneliness and the study of the overall field of tools and strategies of loneliness informatics was carried out. The need to study loneliness in younger people is addressed and particular digital solutions and interventions developed for younger people are presented. This paper can be used to overcome the challenges of technological gaps in the studies and strategies developed for loneliness. The findings of this study show that the majority of interventions and reviews are focused on older people, with ICT-based and social media–based interventions showing promise for countering the effects of loneliness. There are new technologies, such as conversational agents and robots, which are tailored to the particular needs of younger people. This literature review suggests that the digital solutions developed to overcome loneliness can benefit people, and younger people in particular, more if they are made interactive in order to retain users.

## Introduction

Loneliness is a global health epidemic that affects a significant number of global populations. In the United States, an estimated 17% of adults aged 18 to 70 years report loneliness. Monetary loss as a result of loneliness is estimated to be between US$8074.80 and US$12,0777.70 per person per year in the United Kingdom [1]. The monetary cost of lost days and loss in productivity is estimated to be US$3.14 billion per year for employees in the United Kingdom. Loneliness is also linked to a 30% increase in heart disease, stroke, dementia, depression, and anxiety [2-4]. Loneliness and isolation are interlinked yet separate concepts. Loneliness is defined as the subjective difference between a person’s desired and actual social contact and relations [5]. Loneliness can be subjective, while isolation, on the other hand, is a social phenomenon in which there is actual absence of social engagement and contact both with the immediate family and larger community [6]. This can be acutely the case for older adults, where one-third and one-quarter of them will experience feelings of loneliness and isolation, respectively [7,8].

Social isolation results in the loss of self-esteem and self-confidence and hence the ability to form meaningful social relationships. Stigma may be particularly significant for certain age groups, such as young people [9]. Studies have linked loneliness and social isolation to numerous determinants, such as possible psychological, social, neurocognitive, and genetic mechanisms, as well as relationships with community and social factors [9]. What makes loneliness hard to study as a category is that it can be viewed as a transdiagnostic construct that can occur alongside, as well as cause and predict, a variety of mental health conditions [10]. Transient loneliness results in emotional distress, which can be caused by social disconnection but can be coped with. The problem arises if loneliness persists in the long-term, at which point it can result in the altering of neurobiological and behavioral patterns. Cacioppo and Hawkley [11] theorize about a self-enforcing loop of chronic loneliness, leading to an increase in hypervigilance and cognitive bias toward social threat, hence eliciting hostile behavior toward social interaction.

There have been several systematic and scoping reviews done to understand loneliness. Similarly, from a health informatics perspective, there have been multiple systematic reviews of the connection between loneliness and mental health [6,8,12-14]. From the health informatics side, there also have been studies that deal with the application of technology-based interventions to cope with loneliness. There are also scoping reviews to find the effectiveness of technology-based intervention strategies for loneliness [14]. However, there are limitations to the scoping reviews done on loneliness, both theoretical (ie, psychological aspect) and technical (ie, from a health informatics perspective). The first limitation is that almost all of them focus on intervention strategies for older people. Second, from a health informatics perspective, a comprehensive study of technology-based interventions has not been carried such that a general overview of different technologies used to cope with loneliness can be drawn.

Young people are particularly prone to being lonely. The reasons for this are multifaceted. The first context is a developmental one, wherein adolescent social interaction is key to identity formation and individuation from family [15,16]. Any negative experience as to social interaction can result in self-imposed isolation, and thus loneliness. The other context is that of increased risky behavior, which can be exacerbated by the feeling of loneliness. Studies have shown that loneliness in young people results in increased risks of smoking, taking drugs, and consuming alcohol [17]. Studies have also shown that young people are particularly prone to mental health problems, with 1 in every 8 young people aged 5 to 19 years having a mental health issue [18]. Because loneliness and mental health problems are related in adults and 75% of all mental health problems emerge before the age of 24 years, it is important to study loneliness in younger people and overcome the condition earlier to avoid consequent mental health problems.

Finally, the COVID-19 pandemic has had a greater impact on the feeling of loneliness among younger people than the general population, with 50.8% of people aged 16 to 24 years reporting feeling lonely as compared to the general population ratio of 30.9% [17]. Other research has shown an increase in feelings of anger and perceived stress during the COVID-19 pandemic as compared to prepandemic levels [19].

These factors make studying loneliness in young people urgent. A review is important to find out what technology-based interventions are available for loneliness among young people and what kind of interventions will be more effective than others in reducing loneliness among young people. This paper was written after consulting a wide range of resources on loneliness, from theoretical understandings of loneliness to technological interventions for overcoming loneliness. The resources and papers were found using Google Scholar. No particular inclusion methodology was used to review or study papers other than to find papers that gave an introduction to both loneliness on a theoretical level and for technological interventions. The available literature was further studied to identify different categories of technological interventions to overcome loneliness. The survey of available literature revealed that a relatively smaller number of technological interventions focus on overcoming loneliness in younger people than in older people. Summarily, there are 3 types of technology-based interventions in the literature on loneliness in young people. The first type deals with social media–based intervention strategies for fighting loneliness in young people. The second type deals with broader applications of information and communication technology (ICT) ranging from videoconferencing, psychotherapy, and remote counselling. The third type of literature available in loneliness informatics is about exploring the effects of the prevalent use of social media in young people and its relationship with loneliness.

The major contributions of this literature review are as follows:

- Situating the literature on loneliness in young people in loneliness informatics through a short overview of the literature on loneliness across all age groups.
- Comprehensively reviewing technology-based intervention strategies for reducing loneliness in younger people.
- Highlighting gaps in the current literature on loneliness in young people.
- Exploring and proposing methods and technology tools that can be used to build more effective tools for reducing loneliness in younger people.

## Theoretical Understanding of Loneliness

While we provided some definitions of loneliness in the Introduction section, it is imperative for the field of loneliness informatics to be well-connected with the theoretical grounding of loneliness. For this purpose, this section will provide a detailed theoretical understanding of loneliness through different psychological, biological, and social theories.

While loneliness can have positive connotations in philosophical contexts and in some evolutionary theories, the core elements of the concept of loneliness are unwelcome feelings associated with a lack of companionship, either in quantity or quality. Cognitive processes determine how a person processes the feeling of loneliness. Taking this, Paplau and Perlman [20] formally defined loneliness as “the unpleasant experience that occurs when a person’s network of social relationships is deficient in some important way, quantitively or qualitatively.” Loneliness is a multidimensional phenomenon with 3 clear, major components [21]. The first component is deprivation, which can be called the core component. The second component is the temporal dimension, that is, whether the feeling of loneliness is perceived as changeable or hopeless. The third component is that of different types of emotional aspects, such as feelings of sorrow, sadness, guilt, and shame. The deprivation and emotional components of this understanding of loneliness point to the personal and social determinants of loneliness. Thus, loneliness can be the result of emotional isolation caused by the absence of attachment and social isolation caused by the absence of community.

Loneliness is understood by the following three major theoretical frameworks [22]:

1. Social needs perspective: This theory claims a direct relationship between the objective social reality of relationship deficit and the subjective experience of loneliness. When a relationship does not satisfy the desired social needs, loneliness arises. Desired social needs change over time and the adherents of this theory claim that the experiences of loneliness are expected to change over the lifespan [20].
2. Cognitive discrepancy model: This model focuses on a person’s subjective evaluation of the fulfillment relationships bring rather than the social needs fulfilled by the relationships. People tend to judge the satisfaction their relationships bring on their own internal scale. Through this scale, comparison is made with other relationships in their circle or through a perceived sense of other people’s relationships. The cognitive discrepancy arises when the actual relationships do not meet qualitatively with the perceived standard as judged by a subjective internal scale.
3. Evolutionary theory of loneliness (ETL): The previous 2 theories emphasize the social environment and its relationship to the subjective feeling of loneliness. The ETL, on the other hand, focuses both on the social environment and genes in developing feelings of loneliness. According to the ETL, the feeling of being lonely even in the presence of others is a biological warning system, which is found across all species, that signals that the present relationships one finds themselves in are either damaged or threatened. The negative feelings associated with being lonely motivate one to replace the negative bonding or relationships. The ETL states that the feeling of loneliness promotes emphasis on vigilance for social threats and an increased concern for one’s self-interests. This behavior is influenced both by environmental factors and genetic inheritance. Furthermore, according to the ETL, the onset of loneliness is not controlled by one gene but by multiple genes. The role of these genes in determining loneliness varies from one person to the other. For the social component, the ETL claims that the expression of loneliness genes depends on the social environment. Therefore, the variability in feelings of loneliness is determined by the interaction of genes and the social environment. The ETL has multiple components, but for the study of loneliness informatics and technology-based intervention, one component is important. Reaffiliation motive, which is part of the ETL, states that aversive feelings of social isolation caused by loneliness motivate individuals to reconnect with other people [23].

## Situating Loneliness Informatics for Young People: Context for Technology-Based Interventions

This section will give an in-depth literature review of loneliness in younger people and technology-based interventions for loneliness in general. Loneliness in younger people has different social and generational dynamics. Therefore, loneliness in younger people must be understood from its particular dynamics in order to design effective digital interventions. In the first subsection below, the theoretical understanding of loneliness in younger people is given, while in the second subsection, an overall picture of loneliness informatics is given with the aim of situating loneliness informatics for younger people.

### Understanding Loneliness in Younger People

Loneliness in younger people has yet to be explored and tackled proportionately to the prevalence of loneliness in younger people. Population studies of loneliness find a U-shaped age distribution of loneliness; that is, the rate of loneliness is higher in older and younger people. The epidemiological work carried out on loneliness and its health impacts focus more on older people [24-26]. The findings as well as theoretical underpinnings of the works that focus on older people cannot be directly linked and applied to younger people as the dynamics of loneliness vary by age and cultural contexts and groups. Weiss [27] suggested that the primary function of young adulthood is to shirk off parental attachments and to make life and social connections according to one’s own subjective perceptions and interests. Young adulthood is also the age when the new life of college begins, which also brings with it the experience of social distance from the established social networks.

The reported evidence about loneliness in younger people suggests an increased occurrence of loneliness in adolescence years. Younger people and children aged 10 to 24 years report loneliness as a feeling of isolation and a sense of exclusion and disconnection from their social context [28]. It was also noted by the UK Office of National Statistics that people aged 16 to 24 years were the loneliest of all age groups [28]. It was shown by Qualter et al [29] that chronic loneliness predicts adolescent depression. Loneliness affects the quality of life particularly because of the stigma associated with seeking formal or informal help [30]. The loneliness reported by younger people is even higher than that reported by older people, as reported by some studies. A web-based survey found that out of 55,000 people, 40% of people aged 16 to 24 years reported loneliness as compared to 27% of people older than 75 years [28]. Behavioral genetic analysis indicates that loneliness and depression are influenced by the same genes, while the effects of these genes can be higher in younger people. In view of the high prevalence of loneliness in younger people and its potential lifelong consequences, it is paramount to gain a better understanding of loneliness and its relation to depression in younger people and to design age-appropriate treatments.

Psychological interventions for overcoming loneliness have proven to work if the target areas are changing maladaptive social perception, increasing social contact, increasing opportunities for social interaction, and improving social interaction and interpersonal skills [31]. Technology-based interventions for loneliness focus on one or more of these target areas. Technology can engulf different modes of interventions, such as individual psychotherapy with cognitive behavioral therapy, mindfulness therapies, and social support groups [32-34]. Because of the COVID-19 pandemic, remote psychotherapies and support groups have been used. Internet-based cognitive behavioral therapy and skills training to access internet and web-based support have been used for remote therapies [35,36].

**Figure 1:**
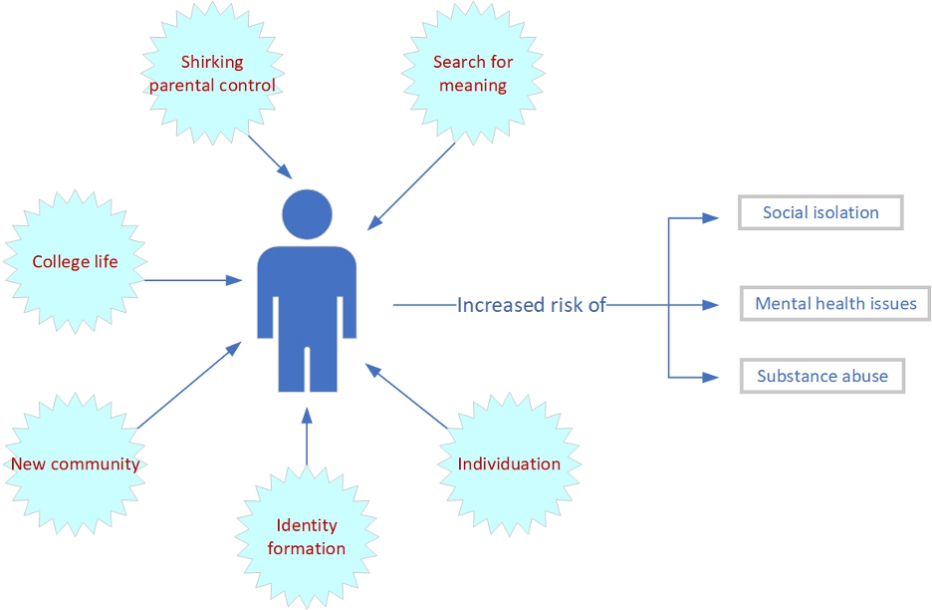
Dynamics of loneliness in young people.

Figure 1 presents the particular dynamics of loneliness in younger people and shows that the causes of loneliness in younger people are distinct; therefore, the solutions for overcoming loneliness in younger people should address these distinct causes. These distinct causes include processes of identity formation, individuation from family, as well as experiencing new environments, such as that of college and a new city [15,16]. Figure 1 has presented these parameters in graphical form with the consequences of increased loneliness. The increased risk of mental health issues, isolation, and substance abuse are presented [18,37].

### Situating Loneliness Informatics: Overview of the Literature

This section aims to provide a brief overview of studies and technology-based interventions for areas and concerns other than loneliness in younger people.

There have been scoping reviews done for ICT-based programs and interventions for older people. A scoping review of reviews was carried out to study the effectiveness of communication technologies to reduce the feeling of loneliness in older people [14]. The review included 28 studies, which combined covered 248 primary studies spanning over 50 years. The conclusion was that communication-based technologies do in fact reduce feelings of loneliness in older people. Although, the study also found that there was a lack of evidence and limited insight into innovative technologies, such as extended reality frameworks. In work by Shah et al [1], a systematic review and meta-analysis was carried out for digital technology–based interventions to reduce loneliness in older adults. The study analyzed 6 articles including a total of 646 participants. The study showed no statistical difference between the effectiveness of digital intervention, but it self-reported the lack of enough studies and small sample size of participants as the cause for the lack of validating effectiveness.

However, there are studies (eg, [38]) that establish the relationship between communication-based technologies and reduction in the feeling of loneliness. A cross-sectional study of 4315 adults older than 50 years reported that rural older adults who used technology less frequently felt loneliness more than urban older adults. The study also explored the relationship between race, urbanity, and age, and recommended that technology-based intervention be consider a social technology to address rural and racial disparities. Nevertheless, the connection or direct correlation between social media technology use and reduction in the feeling of loneliness is not strongly established. Wiwatkunupakarn et al [12] performed a study of the effectiveness of social networking site usage in older people for reducing loneliness. The study analyzed 10 observational studies and 5 experimental studies, of which 5 studies focused on loneliness and social isolation. Among the observational studies, some evidence was found that the use of social networking sites was associated with reductions in the feelings of loneliness and depression; however, the study was lacking on the experimental side. The conclusion here, too, required more studies to establish unambiguously the relationship between technology use and loneliness.

As can be seen from the reviews presented in this section, work in technology-based intervention to reduce loneliness has been focused on older people. There is a protocol design study for carrying out technology-based interventions for older people, which details the parameters on which such a scoping review can be done [6]. There is also a survey study about designing a digital psychoeducation tool for reducing loneliness in older people [39]. The study highlighted how the older adults’ concerns about technology could be incorporated through an iterative process in a co-design approach to designing a technological approach. Budak et al [40] studied a technology-based intervention for dementia and loneliness with a particular focus on the role of assistive technologies. It was concluded that assistive technologies meant to fight dementia have a positive effect in reducing loneliness in older people.

Many studies meant to analyze loneliness in young people also consider older people. For example, Loveys et al [31] found that technology-based interventions for both older and younger people were acceptable to both groups. Other studies also focused on surveying and designing feasibility cases for technology-based intervention in older people [41,42]. The common denominator in these technology-based studies is that both older and younger people are considered.

## Loneliness Informatics for Younger People

This section presents in detail the literature on loneliness informatics for younger people. This section aims to present focused technological interventions for younger people in order to build upon them. The following subsections discuss each in detail.

### ICT-Based Interventions

While ICT-based interventions are used extensively for overcoming loneliness in older people in the literature, it can also be applied to younger people. ICT-based interventions for loneliness in older people suit their needs of communication and connection as their loneliness is often born out of social isolation. Pitman et al [9] presented a scoping review of reviews of ICT-based interventions for older people; however, such reports do not exist for younger people.

Studies on the effectiveness of ICT-based interventions for young people are rare. Internet, videoconferencing, email, telephone, video game consoles, tablets, and smartphones are the mediums for implementing ICT-based loneliness intervention strategies. The use of these mediums and tools is prevalent in younger people, and intervention strategies for loneliness for younger people can be designed around them. In work by Stephens-Reicher et al [43], an evidence base was built to see the efficacy of ICT interventions for youths facing social isolation and ensuing mental health problems. It has to be noted that social isolation is not totally specific to young people, and other groups, such as transgender people, may feel it and may rely upon the internet to find safety and connection. Similarly, for young people experiencing limited career opportunities as well as limited social and educational opportunities, ICT tools may be the gateway to building friendships and connection and seeking ways for growth. It was also reported by Yeo and Sawyer [44] that young people with chronic illnesses may have limited opportunities for social interaction, which can be overcome by the use of ICT for continual social and educational participation [45].

The social isolation born out of geographical location and socioeconomic parameters, such as race, class, and religious identity, can reduce access to face-to-face services for loneliness and other mental health–related services. ICT-based interventions for loneliness and related mental health problems, if delivered strategically, can overcome geographic and socioeconomic discriminations. The developmental challenges faced by youths that lead to social isolation, such as growth and consolidation of identity, maturation of identity, and transition into formal schooling, can well be addressed by ICT-based interventions. The specific importance of social and digital media in web-based spaces for providing support during the COVID-19 pandemic has been reported [46]. The ability to connect with others is also reported to be associated with well-being achieved through using communication technologies [47]. The positive effects of technology intervention were reported to be 44% [29]. A scoping review of interventions specifically for youths suggested that technology may be an effective alternative to face-to-face interventions as 90% of younger adults use the internet at least occasionally [48]. The current literature is mostly focused on exploring technology-based interventions to overcome loneliness for older adults. This calls for the need to explore ICT and other technology-based interventions to overcome loneliness in younger people.

### Social Media–Based Interventions

Like ICT-based interventions for loneliness, social networking sites (SNSs) and social media–based interventions for loneliness have been considered for older adults in the literature. Because of the wide appeal and use of social media and SNSs, there are a few studies of these mediums for loneliness interventions in younger people as well. The main distinction between social media and SNSs is that SNSs allow users to share content with limited users to see responses from friends, family members, or followers [12,49]. This makes popular social media sites, such as Twitter, Facebook, and Instagram, SNSs but not the other way around. SNSs with limited circles of interaction were found to be effective in social support and be a source of health information [50]. Loneliness is reported to be reduced through the use of SNS-based interventions for older people [51].

Social media–based interventions for loneliness can address the needs of younger people to connect and create bonds. The displacement of identity and social relations, which result in loneliness in younger people, can be overcome through SNS-based interventions. Studies show that social media technology enables personal relationships inside and outside of one’s social circle. The use of social media is also shown to result in a deepened sense of identity and purpose [52,53]. When considering the use of social media–based interventions for youths, the following questions should be addressed: Does social media help in forming social relationships among youths or the opposite? Do some youths, on the basis of their socioeconomic or geographical realities, become more prone to using social media as their primary source of socialization? Finally, is social media use associated with problematic internet use (PIU), and at what point do the benefits of social media use change into PIU?

A study in 2018 explored the relationship of social media use over time in youths and frequent face-to-face communication with close social circles, such as family and friends, and their associated subjective well-being [54]. The research provided little support for the social displacement model theory, which states that social media displaces or replaces real-life social relationships, which have depth with remote social media connections. In fact, the study found a positive association between social media use and well-being changes. Similarly, there is little support for the assertion that increased social media use can result in loneliness and depression [55]. If social media and the internet are better integrated into the lives of the participants, such as when they are used to interact with friends and family, the result is the feeling of loneliness fades over time.

The social compensation model, contrary to the social displacement model, asserts that social media may reduce the feeling of loneliness because it may be seen by users as a safer space for exploring social connections. Ellison et al [56] found a positive correlation between the self-esteem of college students and the use of social media sites. Individuals who are socially anxious, introverted, or less likely to self-disclose may find social media particularly helpful in establishing contacts, thus reducing the feelings of social isolation and loneliness. The self-disclosure hypothesis has also been supported in the literature [57]. It was showed that, because of reduced visual, auditory, and contextual signals, the users may become more confident to share their feelings, vulnerabilities, and emotions as they perceive the medium and the interaction to be less judgmental, hence leading to more self-disclosure and deeper relationships.

In a report by Vincent [58], the use of social media to develop a sense of belonging was studied. Belonging and loneliness are associated—a sense of belonging to a community brings down the feeling of loneliness. The study explored the role of social media as a therapeutic model used by college counsellors. The results showed that such use of social media increased the sense of belonging. This study suggests that social media can be used by college counselors as a potential tool to overcome loneliness in college students. Similar results were reported by Liu et al [59] for minority youths to identify more with web-based friends and to report more support on the internet than in real life. When belonging and overcoming loneliness is the central motivating factor behind the use of social media, it was found that the sense of community is likely to increase, resulting in a reduction in loneliness [60].

### Robots, Conversational Agents, and Digital Humans: Application to Loneliness in Younger People

Robots are another area of technology-based interventions for loneliness. Robots are particularly useful for older adults [31,61]. Studies have suggested different robots for companionship and reported a general reduction in loneliness and an increase in the sense of companionship [46,62-64]. Social robots are also reported to improve loneliness in younger adults [65]. Similarly, conversational agents (CAs), where robots or interactive mobile or software applications conversate, have been effective in older people, especially if they are human-like embodied and use proactive communications.

CAs can be employed for all age groups as they respond to the needs of people with loneliness. Digital humans (DHs) can be classified as a type of CA, with the difference that the agent who is interacting with the user has a human-like appearance and uses artificial intelligence to build real-time social and emotional engagement with users [66]. Interacting with such an agent can reduce the feeling of loneliness, as the appearance of DHs is human-like because they are based on real-life characters. Some DHs are designed to include a complex cognitive architecture modelled on human behavior, which is able to show attachment and separation toward users to influence their behavior. DHs can be useful in delivering remote loneliness intervention as they are scalable and require access to the internet and a device, be it a computer, tablet, or mobile device.

The features of DHs that can be useful for countering loneliness are the real-life tasks that can be recommended by the human-like CA to the user. In a study by Loveys et al [31], a DH was designed by the name of “Bella”. Bella was modelled to be a woman of Maori and New Zealand European decent, and the program was accessible through the web. The conversation facilitator (ie, Bella), responded to users’ input through speech, text, or a prompt to press a button on a screen. Bella engaged in human-like facial gestures, and her face portrayed joy and concern. Bella also had linguistic variation of her own such that the user did not feel mechanical repetition of the same phrases. The relationship-building strategies employed by Bella were taken from psychology and human-computer interaction research [67]. These included tasks such as positive self-affirmation, reaching out to a friend, and complimenting someone you know. Participants who took part in interactions with Bella reported that Bella was useful in improving the feeling of loneliness and that they would like to interact with Bella again in the future. The study also reported that it was easy to train younger people to use the service either in a clinic room at the university or through video calling, and technical support requests were low for younger people during the training. DHs are used for a variety of purposes. DHs play a key role in augmenting reality and in other forms of extended reality. These are not limited to mental health applications, or in the case of this paper, to loneliness informatics.

## Conclusion and Future Directions

This paper presented a comprehensive review of loneliness informatics. While there has been significant work done on countering loneliness in older adults, the same is not true for younger people, who have the highest loneliness numbers. This paper discussed in detail the particular dynamics of loneliness in younger people, which range from developmental changes to transitioning into new social contexts to cultural and psychological dynamics that begin to form at adolescence, such as the search for personal identity, meaning, and purpose. This paper presented an overview of loneliness informatics for older people, which included the use of digital media, social media, and robotics for interventions to cope with loneliness.

The focus of loneliness informatics is not on the younger population. However, in terms of social media, there have been some studies carried out. One downside of social media and digital media use is that it is often asserted that they are responsible for increased loneliness in younger people. The reported studies in this paper suggested that this may not be the case. However, there still is the possibility of PIU. There are factors that may affect addiction possibilities, such as low social capital, which can carry over to social media and bullying, which can happen in social media and web-based spaces. Some students and younger people who have social anxiety issues use social media for socialization. Overall, social media was found to be an effective intervention tool and strategy for countering loneliness. CAs and DHs, forms of digital technology with the added features of human-like language processing or human-like appearance, were also found to be helpful as coping mechanisms for loneliness. Because of the requirement of technology competency, the study found that they are more helpful to younger people.

Understanding loneliness from a health informatics perspective is an open field. There are issues, gaps, and challenges that should be addressed in future works. A comprehensive review of different aspects of the application of technology to understand loneliness and to design intervention programs for overcoming loneliness can be undertaken. Examples of such reviews can be the application of virtual reality– and extended reality–based interventions for loneliness, machine learning–based applications in serious games for loneliness, and comparative analysis of these technologies for younger and older people. There are also other aspects of loneliness and its intersection with technology that need to be explored, such as whether the use of technology, especially social media, leads to loneliness and how to use social media effectively for countering loneliness. Other than analysis of the literature, loneliness informatics can use data to understand loneliness in different geographical regions as well as different contexts. One challenge to loneliness informatics is the availability of data, which needs to be approximated from different sources, such as social media and news analyses. Another challenge is that the intervention strategies have to consider the social and economic contexts of loneliness, which need policymaking at multiple levels of government and organizations.

## Abbreviations

CA: conversational agent
DH: digital human
ETL: evolutionary theory of loneliness
ICT: information and communication technology
PIU: problematic internet use
SNS: social networking site
